# Bullying and co-occurring psychological distress, self-harm and attempted suicide in adolescents – health inequalities by sexual identity

**DOI:** 10.1093/pubmed/fdag038

**Published:** 2026-05-15

**Authors:** Amal R Khanolkar, Jayati Das-Munshi, Laia Becares

**Affiliations:** Department of Population Health Sciences, School of Lifecourse and Population Sciences, King’s College London, United Kingdom; Department of Psychological Medicine, Institute of Psychiatry, Psychology and Neuroscience, King's College London, United Kingdom; Population Health Improvement (PHIUK), United Kingdom; King’s College London ESRC Centre for Society and Mental Health (CSMH), United Kingdom; Department of Global Public Health, King’s College London, United Kingdom

**Keywords:** mental health, children, epidemiology

## Abstract

Sexual minority youth (bisexual, gay and lesbian individuals) consistently report worse mental health and higher levels of bullying compared to heterosexual peers. Studies have largely reported these associations with individual mental health problems. No study has examined associations between past experiences of bullying and co-occurring common mental health problems and whether these associations differ by sexual identity. In a nationally representative sample of adolescents aged 17 (N=9,767, female:51%, sexual minority:20%), we examined associations between sexual identity and risk for self-reported co-occurring mental-health (MH) problems (psychological distress, self-harm, and attempted suicide). We further examined whether sexual minority adolescents with past experiences of bullying (at ages 11/14) were more likely to report co-occurring-MH compared to peers without bullying. Bisexual (RRR: 9.42, 95% CI: 6.53-13.58) and gay/lesbian (RRR: 6.33, 3.52-11.47) adolescents had higher risk for co-occurring-MH than heterosexual peers. However, sexual minority adolescents (15%) with past bullying were more likely to have co-occurring-MH compared to sexual minority (4%) and heterosexual (2%) peers without bullying.

Sexual minority youth with past experiences of bullying are 7-8 times more likely to report co-occurring-MH problems compared to heterosexual peers without bullying. More effective public health policies are required to reduce the deleterious impact of bullying on mental health.

## Introduction

Sexual minority youth (SMY), i.e. individuals identifying as lesbian, gay, bisexual and mainly heterosexual, consistently report 3-4 times higher levels of psychological distress, self-harm (non-suicidal), and attempted suicide compared to heterosexual peers.^1–3^ Several risk factors are associated with worse mental health including living in heteronormative environments, rejection, stigma and discrimination associated with sexual minority identities.^4^ Relatedly, SMY also report significantly higher levels of bullying than heterosexual peers, which is associated with worse mental health, including self-harming behaviours.^5^^,^^6^ A systematic review including over 300,000 young SMY individuals found that prevalence of bullying was substantially higher in SMY who reported self-harm/attempted suicide compared to SMY and heterosexual peers who did not.^5^

Most studies have separately examined associations between sexual identity and risk for psychological distress, self-harm and attempted suicide which are the most common mental health problems in SMY. However, these three mental health problems have a high risk to co-occur together with subsequent short- and long-term consequences across the lifecourse. Identifying individuals at risk of ≥2 of these common co-occurring mental health problems and the impact of modifiable risk factors like bullying can aid in reducing mental health inequities.

In a nationally representative UK-sample, we examined whether 1) SMY are more likely to report co-occurring psychological distress, self-harm, and attempted-suicide and 2) Co-occurring psychological distress, self-harm, and attempted-suicide are more common in SMY reporting past experiences of bullying, and in comparison, to SMY and heterosexual peers with and without bullying.

## Methods

This study used data from the UK-wide age 17 sweep of the Millennium Cohort Study (MCS). Sexual identity (original categories in Supplemental Table S1) included four categories 1. Completely heterosexual, 2. Mainly heterosexual, 3. Bisexual and 4. Mainly and completely gay/lesbian (hereafter gay/lesbian), was self-reported. Those who did not know/report or with ‘other’ sexual identities were excluded.

Mental health (MH): Psychological distress (symptoms of depression/anxiety) in the previous six months was assessed using the self-reported Strengths and Difficulties Questionnaire emotional symptoms subscale (SDQ-E) comprising five questions. Total scores (range 0 to 10) were categorised into a binary variable indicating ‘close to average’ (<6) vs ‘high/very high levels’ (≥6) of psychological distress (emotional difficulties), using a validated cut-off.^7^^,^^8^ The SDQ-E is validated against diagnostic measures of depression or anxiety disorders and routinely used to capture symptoms of depression/anxiety in the general population and clinical settings.^9^ It is an established screening tool to distinguish those individuals who meet the diagnostic criteria for depression and anxiety from those who do not. Self-harm actions (like cutting/stabbing, burning, bruising/pinching, taking an overdose of tablets and pulling out hair) in the previous year were assessed and a binary variable was created to indicate none vs any kind of self-harm. Attempted suicide was assessed by the question: *“Have you ever hurt yourself on purpose to end your life?”* (i.e., lifetime). Responses were categorised as no vs yes.

### Co-occurring mental health-indicator

Responses from the three individual MH variables were combined to create a categorical variable grouping participants into one of the following categories indicating MH status: (1) No mental health problems, (2) High psychological distress, (3) High psychological distress and self-harm *or* High psychological distress and attempted suicide, (4) Self-harm only *or* attempted suicide only and (5) High psychological distress, self-harm & attempted suicide, i.e., having all three MH problems.

Experiences of bullying were ascertained at ages 11 and 14 (*‘How often do other children hurt you/pick on you on purpose?’* and *‘How often have other children sent you unwanted or nasty emails, texts, or messages, or posted something nasty about you on a website?”*). Answers to both questions were combined to create a binary variable indicating no vs any experience of bullying. Supplemental Table S2 provides a full description of MH, bullying and sexual identity variables.

### Confounders

Included sex assigned at birth (male or female), hereafter sex, ethnicity (White or ethnic minority) and parental income (quintiles). These are established confounders associated with both mental health and SM identity in adolescents.

The study sample included 9 767 individuals with data on sexual identity at age 17. Missing data on covariates were addressed using multiple imputation (explained in detail in Supplemental Methods).^10^

### Statistical analysis

We calculated the frequency of the MH-co-occurring outcome variable by sexual identity. We then examined associations between sexual identity and probability for co-occurring-MH using multiple multinomial logistic regression models. Models were run unadjusted, with mutual adjustment for all three confounders, and additionally adjusted for bullying (to assess whether bullying partly explains associations between sexual identity and co-occurring-MH).

Second, we used multiple multinomial logistic regression to assess risk for co-occurring-MH in relation to sexual identity and bullying (including interactions terms between these two variables to assess variation in probabilities for co-occurring-MH by the different levels of sexual identity and bullying). Adjusted predicted probabilities of co-occurring-MH for all possible combinations of sexual identities and bullying experiences (i.e. 40 categories generated by the interaction terms between the two variables) were estimated using the *‘margins’* command in Stata. Predicted probabilities were visualised (by creating interaction plots using the *‘marginsplot’* command) by past bullying status to aid understanding of interaction terms.

All models were weighted with birth sweep non-response weights to account for the stratified cluster design of the MCS and attrition over time. Analyses were conducted in Stata V18 (StataCorp LP, College Station, Texas).

## Results

**Figure 1:**
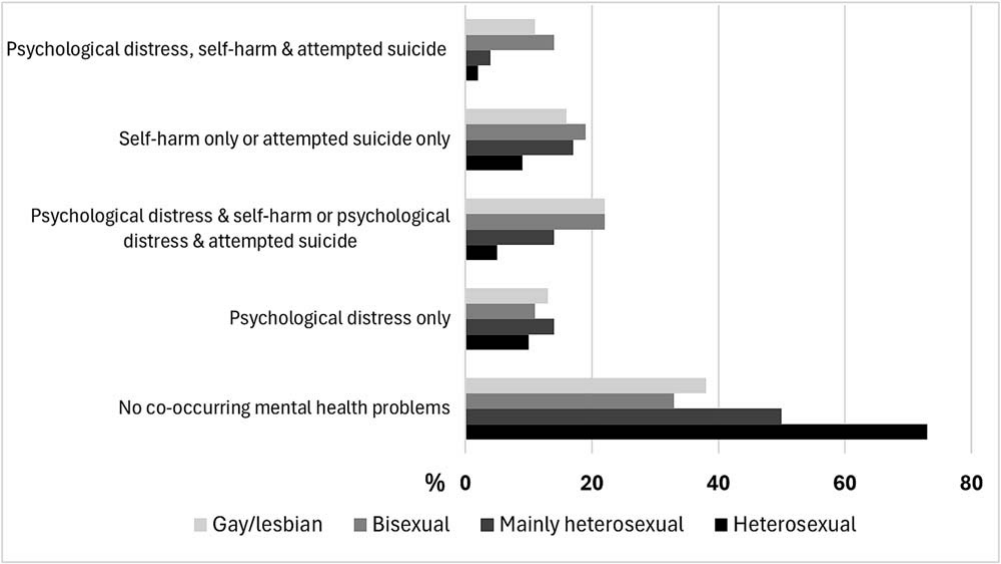
Prevalence of co-occurring mental health problems by sexual identity in 9,767 adolescents aged 17 years from the millennium cohort study footnote: Estimates presented in Supplementary Table S3.

Prevalence of having *no MH problems* was ≤50% in SMY (e.g., 50% in mainly heterosexual, 33% in bisexual and 38% in gay/lesbian adolescents) compared to heterosexual peers (73%), Figure 1 and Supplemental Table S3. All SMY groups had 3-4 times higher prevalence of co-occurring high psychological distress and self-harm/attempted-suicide, increasing to 5-7 times higher prevalence of all three co-occurring-MH problems in bisexual and gay/lesbian groups, compared to heterosexual peers.

All SMY groups had significantly higher risk of individually occurring (e.g., high psychological distress: relative risk ratio [RRR] 1.75, 95% CI 1.38-2.21 for mainly heterosexual, RRR 1.55, 1.09-2.19 for bisexual, RRR 2.12, 1.35-3.33 for gay/lesbian compared to heterosexual peers) *and* co-occurring-MH problems (e.g., high psychological distress and self-harm/attempted-suicide: RRR 3.17, 2.43-4.13 for mainly heterosexual, RRR 6.37, 4.82-8.41 for bisexual and RRR 6.43, 4.36-9.47 for gay/lesbian individuals, Supplemental Table S4). However, the highest RRRs were observed for all three co-occurring-MH problems (e.g., RRR 9.42, 6.53-13.58 for bisexual and RRR 6.36, 3.52-11.47 for gay/lesbians individuals). Bullying was independently and substantially more likely to be associated with the co-occurring of all three MH problems (RRR 4.22, 3.12-5.69) than individual occurring mental health problems (psychological distress: RRR 1.41, 1.16-1.72 and self-harm/attempted-suicide only: RRR 1.65, 1.34-2.03).

**Figure 2:**
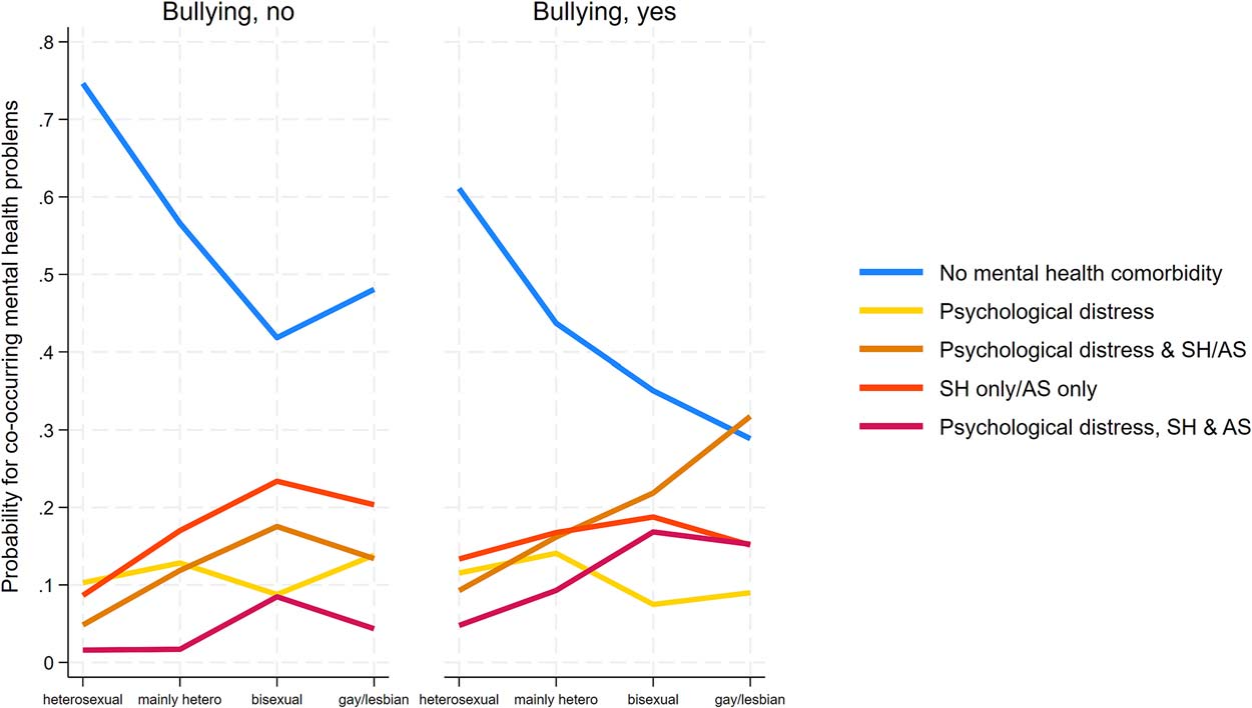
Predicted probabilities for co-occurring mental health problems based on sexual identity and past experiences of bullying in 9,767 adolescents aged 17 years from the millennium cohort study. Footnote: SH: Self-harm, AS: Attempted suicide. Probabilities are based on estimates from multinomial logistic regression models including interaction terms between sexual identity and bullying.

There were minor or no differences in individually occurring mental health problems (high psychological distress only, self-harm only or attempted-suicide only) between those with and without bullying (Figure 2 and Supplemental Figure S1 and Table S5). An exception was higher probability for self-harm or attempted-suicide only among heterosexual adolescents without (5%) and with (9%) bullying. Individuals with bullying had higher probabilities for co-occurring psychological distress and self-harm/attempted-suicide than those without bullying. Further, probabilities for co-occurrence were higher in gay/lesbian individuals with (32%) and without (13%) bullying compared to heterosexuals, both with (9%) and without (5%) past bullying. Similarly, heterosexual adolescents with bullying were >twice as likely (5%) to have all three MH conditions compared to peers without (2%) bullying. These differences in probabilities further increased and were also wider between SMY adolescents with and without bullying (mainly heterosexual: 2% vs 9%, bisexual: 8% vs 17%, gay/lesbian: 4% vs 15% for those without and with bullying, respectively).

## Discussion

SMY are substantially more likely to report to co-occurring-MH problems compared to heterosexual peers. Past experiences of bullying are significantly associated with greater probability for co-occurring high psychological distress, self-harm & attempted suicide in adolescents irrespective of sexual identity. However, the impact of bullying on co-occurring-MH problems appears to be worse in SMY with past bullying compared to SMY and heterosexual peers with and without bullying. This study highlights long-term consequences of bullying across mid to late adolescence and substantial health inequities by sexual identity.

Main strengths of this study included self-identified sexual identity, differences by sexual minority-subgroup, a large nationally representative sample of adolescents in the UK which included the most common mental health problems experienced by UK youth. Some participants may not want to disclose past experiences of self-harm and attempted suicide which might underestimate their prevalence in the MH-co-occurrence variable. Lifetime measure of attempted suicide could impact the temporal assessment of MH as psychological distress and self-harm are measured at age 17. Sexual identity was measured at age 17 but most adolescents are aware of their sexuality much earlier in age.^11^ While sexuality precedes the self-harm and psychological distress it might coincide with bullying in early adolescence. Further studies with clearer temporality between sexual identity, bullying and MH outcomes are needed. These can also examine in greater detail the role of bullying as both a moderator and/or mediator between sexual identity and mental health. Further studies should examine associations by types and frequency of bullying, and differences by gender, ethnicity and socioeconomic deprivation (not possible here due to smaller numbers of participants who are both sexual and ethnic minority and/or deprived). Other common and probable co-occurring mental health problems in young people like eating disorders and ADHD should be studied. The role of SM-specific experiences like disclosing sexual identity, access to support in different environments (home, school and public spaces) and how these might change the impact of bullying on mental health in SMY needs further examination, however such data is often not available in routinely conducted research studies.

Our previous study also using the MCS reported that bullying was significantly more common in SMY compared to heterosexual peers.^10^ While the impact of bullying on mental health is well documented, this is the first study to examine longitudinal associations with co-occurring mental health problems including self-harming behaviours by sexual identity in a nationally representative sample. In 2023, nearly 25% of 17 to 19-years-old in England had a probable MH disorder.^12^ Given that bullying among school-aged children is common,^13^ stronger interventions are required to reduce all forms of bullying including SM victimisation. Further, creating environments of trust and recognising the unique aspects and risks of being sexual minority (like understanding difficulties with sexual-identity disclosure, not subscribing to heteronormative or peer-normative behaviours), ensuring that parents, staff and clinicians are aware of the deleterious impacts of bullying in sub-populations can potentially reduce co-occurring mental health in those experiencing bullying.

## Supplementary Material

Bullying_SM_study_Supplemental_Data_JPH_FINAL_fdag038

## Data Availability

Data for this study is primarily from the age 17 sweep of the Millennium Cohort Study. Data on bullying is from the ages 11 and 14 sweeps of the Millennium Cohort Study. Data can be accessed from the UK Data Service website: https://ukdataservice.ac.uk (after registration and agreeing to licence terms and conditions). Syntax/code used for the analysis in this study is available on request from the corresponding author.

## Notes

**Amal R Khanolkar**, PhD, Researcher

**Jayati Das-Munshi**, PhD, Professor

**Laia Becares**, PhD, Professor
