# Supplementary material for: Bullying and co-occurring psychological distress, self-harm and attempted suicide in adolescents – health inequalities by sexual identity
Source: J Public Health (Oxf). 2026 May 15;48(2):444–8. doi: 10.1093/pubmed/fdag038 (PMC13223567; doi:10.1093/pubmed/fdag038)
Supplement: Bullying_SM_study_Supplemental_Data_JPH_FINAL_fdag038 [file bullying_sm_study_supplemental_data_jph_final_fdag038.pdf]

Bullying and co-occurring psychological distress, self-harm and attempted suicide in adolescents – Health inequalities by sexual identity

Amal R. Khanolkar[1], Jayati Das-Munshi[2] and Laia Becares[3]

1.Department of Population Health Sciences, King's College London, United Kingdom, 2. Department of Psychological Medicine, Institute of Psychiatry, Psychology and Neuroscience (IoPPN), King's College London, United Kingdom & 3. Department of Global Public Health, King's College London, United Kingdom

Supplemental Data File

Supplemental methods – Multiple imputation to address missing data

Like many longitudinal studies, the MCS has experienced attrition over time leading to missing data. We addressed missing data using multiple imputation by chained equations (MICE) assuming data missing at random (MAR) [1]. The MAR mechanism implies that systematic differences between the missing values and the observed values can be explained by observed data. This is a plausible assumption in longitudinal data like the British birth cohorts including the Millennium Cohort Study which collects rich and a wide variety of data from birth. It is reasonable to assume that observed characteristics (like ethnicity and socioeconomic indicators) are independent predictors of missingness. They are also associated with many variables including the wide range of mental and general health outcomes, health behaviours (e.g., smoking, alcohol consumption, drug use, physical activity) of the participants at age 17, and adverse childhood experiences (like parental mental health, parental health behaviours like smoking and alcohol consumption, and bullying) collected across childhood that were included in the imputation model. The imputation model also included BMI from ages 3, 5, 7, 11, 14 and 17, birth weight of study participants, and parental educational level as auxiliary variables to help strengthen the robustness of imputed data. Auxiliary variables with stronger associations with incompletely observed variables or the probability of data being missing, increases the potential for reducing bias [2]. Further, including both baseline and longitudinal variables increases the efficiency of imputing missing data [2]. All participants had data on sex at birth, parental income and ethnicity, and missing data on mental health outcomes was <5% Only bullying had higher missing data at 14.3% Complete cases (i.e., participants with data on all variables) was N=8,136 or 83.3% of the eligible sample. 1,631 (16.7%) individuals were missing data on one or more variables (but largely bullying). We only imputed missing data on mental health indicators and bullying (hence the eligible sample was all individuals with data on sex, ethnicity, parental income and sexual identity). The final study sample was N=9,767 with data on all variables after multiple imputation. Given that missing data for all analysis parameters amounted to <30%, we generated 25 multiple imputations and estimates were combined using Rubin's rules. Our comparison of characteristics, using complete cases and the multiple imputed data (as displayed in Supplementary Table 6), confirmed that the use of multiple imputation was appropriate. Further, comparison of the results obtained from the complete cases and imputed analysis showed that the standard errors were smaller for multiple imputation, demonstrating the efficiency gain of multiple imputation over complete cases analysis.

References:

1. Carpenter JR, Kenward MG. Multiple imputation and its application. Chichester, West Sussex, UK: John Wiley &Sons, 2013.

2. Doidge JC, Edwards B, Higgins DJ, Segal L. Adverse childhood experiences, non-response and loss to follow-up: Findings from a prospective birth cohort and recommendations for addressing missing data. Longitudinal and Life Course Studies. 2017; 8(4):382–400.

Supplemental Table 1. The original categories for sexual identity and ethnicity variables that were combined for analysis

Original categories	Collapsed for analysis
**Ethnicity**	
White	**White**
Mixed	**Ethnic minority**
Indian	
Pakistani	
Bangladeshi	
Black Caribbean	
Black African	
'Other' ethnic group	
**Sexual identity**	
Completely heterosexual/ straight	**Heterosexual**
Mainly heterosexual/ straight	**Mainly heterosexual**
Bisexual	**Bisexual**
Mainly gay or lesbian	**Gay/lesbian**
Completely gay or lesbian	
Other	**Excluded**
Don't know and prefer not to say	**Excluded**

Supplemental Table 2. A detailed description of key variables from the Millennium Cohort Study used in this study

Outcome	Question(s) in cohort member computer-assisted personal interview (CAPI), self-completion interview (CASI) or online questionnaire (CAWI)	Binary or continuous	Comments
**Mental health at age 17**			
Self-reported Strengths and Difficulties Questionnaire (SDQ)<br>- Emotional symptoms subscale	Consists of 5 items or questions:<br><br>-I get a lot of headaches, stomach-aches or sickness<br>-I worry a lot<br>-I am often unhappy, down-hearted or tearful<br>-I am nervous in new situations. I easily lose confidence<br>-I have many fears, I am easily scared	Binary:<br><br>'close to average' (<6) vs. 'high/very high levels' (≥6) of difficulties	Options for each item:<br><br>1. Not true<br>2. Somewhat true<br>3. Certainly true<br><br>Aims to capture emotional feelings (symptoms of depression and anxiety) in the past six months
Attempted suicide	Have you ever hurt yourself on purpose in an attempt to end your life?	Binary:<br><br>No vs yes	Lifetime
Self-harm	During the last year, have you hurt yourself on purpose in any of the following ways?<br><br>Cut or stabbed yourself<br>Burned yourself<br>Bruised or pinched yourself<br>Taken an overdose of tablets<br>Pulled out your hair<br>Hurt yourself some other way	Binary:<br><br>No vs yes (any kind of self-harm)	Past year
Sexual identity at age 17	Which of the following options best describes how you currently think of yourself?<br><br>Completely heterosexual/straight<br>Mainly heterosexual/straight<br>Bisexual<br>Mainly gay or lesbian<br>Completely gay or lesbian<br>Other<br>Don't know<br>Prefer not to say	Categorical:<br><br>-Completely heterosexual (*or* heterosexual)<br><br>-Mainly heterosexual<br>-Bisexual<br>-Mainly & completely gay/lesbian	
Bullying at ages 11 & 14	Ages 11 & 14: How often do other children hurt you/pick on you on purpose?<br><br>Most days<br>About once a week<br>About once a month<br>Every few months<br>Less often<br>Never<br><br>Age 14 only: How often have other children sent you unwanted or nasty emails, texts, or messages, or posted something nasty about you on a website?<br><br>Most days<br>About once a week<br>About once a month<br>Every few months<br>Less often<br>Never	Binary:<br><br>Monthly/every few months/less often/never (rarely or none) vs. most days/weekly (indicating frequent bullying)<br><br>Binary:<br><br>Monthly/every few months/less often/never (less rarely or none) vs. most days/weekly (indicating frequent cyber bullying)<br><br>Final binary variable: Rarely/none vs. any of the three variables above indicating frequent experience of bullying	Period unclear as not indicated in questionnaire but wording suggests in the past few months preceding the interview

Supplemental Table 3. Prevalence of co-occurring mental health problems by sexual identity in 9,767 adolescents aged 17 years from the Millennium Cohort Study

Prevalence of mental health problems by sexual identity	%	95% CI
No mental health problems (67%)		
Heterosexual	73	72-74
Mainly heterosexual	50	47-53
Bisexual	33	3-37
Gay/lesbian	38	32-44
High psychological distress only (11%)		
Heterosexual	10	9-11
Mainly heterosexual	14	12-17
Bisexual	11	8-13
Gay/lesbian	13	8-17
High psychological distress & self-harm OR high psychological distress & attempted suicide (8%)		
Heterosexual	5	5-6
Mainly heterosexual	14	12-16
Bisexual	22	19-25
Gay/lesbian	22	16-27
Self-harm only OR attempted suicide only (11%)		
Heterosexual	9	9-10
Mainly heterosexual	17	15-19
Bisexual	19	16-22
Gay/lesbian	16	12-21
High psychological distress, self-harm & attempted suicide *(all mental health problems, 3%)*		
Heterosexual	2	2-3
Mainly heterosexual	4	3-5
Bisexual	14	12-17
Gay/lesbian	11	7-15

Psychological distress assessed using the Strengths and Difficulties Questionnaire [SDQ]; emotional symptoms subscale (total scores <6=close to average and ≥6=high/very high levels of emotional difficulties).

Supplemental Table 4. Associations between sexual identity, bullying and co-occurring mental health problems in 9,767 adolescents aged 17 years from the Millennium Cohort Study.

	Model 1		Model 2[a]		Model 3[b]	
	**RRR**	**95% CI**	**RRR**	**95% CI**	**RRR**	**95% CI**
No mental health problem (67%)	**Reference**		**Reference**		**Reference**	
High psychological distress only (11%)						
**Sexual identity**						
Heterosexual	1		1		1	
Mainly heterosexual	**2.02**	**1.61,2.55**	**1.77**	**1.40,2.24**	**1.75**	**1.38,2.21**
Bisexual	**2.00**	**1.41,2.83**	**1.58**	**1.11,2.25**	**1.55**	**1.09,2.19**
Gay/lesbian	**2.32**	**1.48,3.62**	**2.23**	**1.42,3.50**	**2.12**	**1.35,3.33**
**Sex**						
Male			1		1	
Female			**3.20**	**2.70,3.79**	**3.29**	**2.78,3.91**
**Parental income**						
Quintile 1			1		1	
Quintile 2			1.19	0.94,1.51	1.18	0.94,1.50
Quintile 3			1.20	0.94,1.52	1.18	0.93,1.51
Quintile 4			1.18	0.93,1.50	1.15	0.90,1.46
Quintile 5			1.16	0.91,1.49	1.12	0.88,1.44
**Ethnicity**						
White			1		1	
Ethnic minority			**0.70**	**0.54,0.90**	**0.71**	**0.55,0.91**
**Past bullying**						
No					1	
Yes					**1.41**	**1.16,1.72**
High psychological distress & self-harm OR high psychological distress and attempted suicide (8%)						
**Sexual identity**						
Heterosexual	1		1		1	
Mainly heterosexual	**3.77**	**2.91,4.88**	**3.25**	**2.51,4.22**	**3.17**	**2.43,4.13**
Bisexual	**8.96**	**6.80,11.79**	**6.82**	**5.14,9.07**	**6.37**	**4.82,8.41**
Gay/lesbian	**7.76**	**5.20,11.57**	**7.37**	**4.93,11.02**	**6.43**	**4.36,9.47**
**Sex**						
Male			1		1	
Female			3.94	3.19,4.87	4.3	3.46,5.33
**Parental income**						
Quintile 1			1		1	
Quintile 2			1.17	0.88,1.57	1.15	0.86,1.53
Quintile 3			**1.38**	**1.07,1.76**	**1.32**	**1.02,1.69**
Quintile 4			1.25	0.93,1.69	1.16	0.86,1.56
Quintile 5			1.13	0.83,1.55	1.02	0.74,1.39
**Ethnicity**						
White			1		1	
Ethnic minority			**0.51**	**0.36,0.70**	**0.53**	**0.38,0.73**
**Past bullying**						

No					1	
Yes					**2.36**	**1.90,2.91**
Self-harm only OR attempted suicide only (11%)						
**Sexual identity**						
Heterosexual	1		1		1	
Mainly heterosexual	**2.46**	**1.94,3.11**	**2.41**	**1.89,3.06**	**2.36**	**1.85,3.00**
Bisexual	**4.34**	**3.28,5.73**	**4.21**	**3.19,5.56**	**4.06**	**3.07,5.35**
Gay/lesbian	**3.46**	**2.16,5.57**	**3.42**	**2.13,5.48**	**3.18**	**1.98,5.11**
**Sex**						
Male			1		1	
Female			1.12	0.96,1.31	1.17	1.00,1.38
**Parental income**						
Quintile 1			1		1	
Quintile 2			1.08	0.86,1.36	1.07	0.86,1.34
Quintile 3			0.85	0.68,1.08	0.84	0.67,1.06
Quintile 4			1.08	0.85,1.36	1.04	0.82,1.31
Quintile 5			0.97	0.75,1.25	0.92	0.71,1.19
**Ethnicity**						
White			1		1	
Ethnic minority			0.87	0.69,1.11	0.89	0.70,1.13
**Past bullying**						
No					1	
Yes					**1.65**	**1.34,2.03**
High psychological distress, self-harm & attempted suicide *(all mental health problems, 3%)*						
**Sexual identity**						
Heterosexual	1		1		1	
Mainly heterosexual	**2.51**	**1.73,3.66**	**2.32**	**1.57,3.43**	**2.21**	**1.49,3.28**
Bisexual	**13.86**	**9.82,19.56**	**10.65**	**7.45,15.23**	**9.42**	**6.53,13.58**
Gay/lesbian	**8.54**	**4.91,14.83**	**7.95**	**4.44,14.24**	**6.36**	**3.52,11.47**
**Sex**						
Male			1		1	
Female			**3.73**	**2.61,5.33**	**4.32**	**3.01,6.21**
**Parental income**						
Quintile 1			1		1	
Quintile 2			1.49	0.93,2.40	1.42	0.89,2.27
Quintile 3			**1.72**	**1.07,2.76**	**1.54**	**0.95,2.51**
Quintile 4			**2.86**	**1.77,4.61**	**2.44**	**1.50,3.97**
Quintile 5			**3.94**	**2.59,6.02**	**3.19**	**2.09,4.88**
**Ethnicity**						
White			1		1	
Ethnic minority			0.32	0.20,0.52	0.36	0.22,0.59
**Past bullying**						
No					1	
Yes					**4.22**	**3.12,5.69**

a: Model adjusted for sex assigned at birth, parental income and ethnicity b: additionally adjusted for bullying. Psychological distress assessed using the Strengths and Difficulties Questionnaire [SDQ]; emotional symptoms subscale

(total scores categorised <6=close to average and ≥6=high/very high levels of emotional difficulties), Text in bold: 95% CIs that do not include 1.

Supplemental Table 5. Predicted probabilities for co-occurring psychological distress, self-harm and attempted suicide based on sexual identity and past experiences of bullying in 9,767 adolescents aged 17 years from the Millennium Cohort Study. Estimates are based on multivariable logistic regression models (adjusted for sex, ethnicity and parental income) and including interaction terms between sexual identity and bullying.

Sexual identity, past experiences of bullying & co-occurring mental health problems	Predicted probabilities (margins)	Standard error	95% CI
No mental health problem (67%)			
Heterosexual, no bullying	0.75	0.01	0.73-0.76
Heterosexual & bullying	0.61	0.01	0.58-0.64
Mainly heterosexual, no bullying	0.57	0.02	0.52-0.61
Mainly heterosexual & bullying	0.44	0.04	0.36-0.51
Bisexual, no bullying	0.42	0.03	0.36-0.48
Bisexual & bullying	0.35	0.04	0.27-0.43
Gay/lesbian, no bullying	0.48	0.05	0.39-0.57
Gay/lesbian & bullying	0.29	0.05	0.19-0.39
High psychological distress only (11%)			
Heterosexual, no bullying	0.10	0.01	0.09-0.11
Heterosexual & bullying	0.12	0.01	0.10-0.13
Mainly heterosexual, no bullying	0.13	0.01	0.10-0.16
Mainly heterosexual & bullying	0.14	0.02	0.10-0.18
Bisexual, no bullying	0.09	0.02	0.06-0.12
Bisexual & bullying	0.07	0.02	0.04-0.11
Gay/lesbian, no bullying	0.14	0.03	0.08-0.20
Gay/lesbian & bullying	0.09	0.04	0.02-0.16
High psychological distress & SH OR high psychological distress & AS (8%)			
Heterosexual, no bullying	0.05	0.01	0.04-0.06
Heterosexual & bullying	0.09	0.01	0.07-0.11
Mainly heterosexual, no bullying	0.12	0.01	0.09-0.14
Mainly heterosexual & bullying	0.16	0.03	0.11-0.21
Bisexual, no bullying	0.18	0.02	0.14-0.21
Bisexual & bullying	0.22	0.03	0.16-0.28
Gay/lesbian, no bullying	0.13	0.03	0.08-0.19
Gay/lesbian & bullying	0.32	0.05	0.21-0.42
SH only OR AS only (11%)			
Heterosexual, no bullying	0.09	0.01	0.08-0.10
Heterosexual & bullying	0.13	0.01	0.11-0.15
Mainly heterosexual, no bullying	0.17	0.02	0.14-0.20
Mainly heterosexual & bullying	0.17	0.03	0.12-0.22
Bisexual, no bullying	0.23	0.03	0.18-0.29
Bisexual & bullying	0.19	0.04	0.12-0.26
Gay/lesbian, no bullying	0.20	0.04	0.12-0.28
Gay/lesbian & bullying	0.15	0.05	0.05-0.25

High psychological distress, SH & AS (3%)			
Heterosexual, no bullying	0.02	0.01	0.01-0.02
Heterosexual & bullying	0.05	0.01	0.04-0.06
Mainly heterosexual, no bullying	0.02	0.01	0.01-0.03
Mainly heterosexual & bullying	0.09	0.02	0.05-0.13
Bisexual, no bullying	0.08	0.01	0.06-0.11
Bisexual & bullying	0.17	0.03	0.12-0.22
Gay/lesbian, no bullying	0.04	0.02	0.01-0.08
Gay/lesbian & bullying	0.15	0.04	0.07-0.24

Psychological distress assessed using the Strengths and Difficulties Questionnaire [SDQ]; emotional symptoms subscale (total scores categorised <6=close to average and ≥6=high/very high levels of emotional difficulties), SH: Self-harm, AS: Attempted suicide.

Supplemental Figure 1. Predicted probabilities for co-occurring mental health problems based on sexual identity and past experiences of bullying in 9,767 adolescents aged 17 years from the Millennium Cohort Study (including 95% CIs).

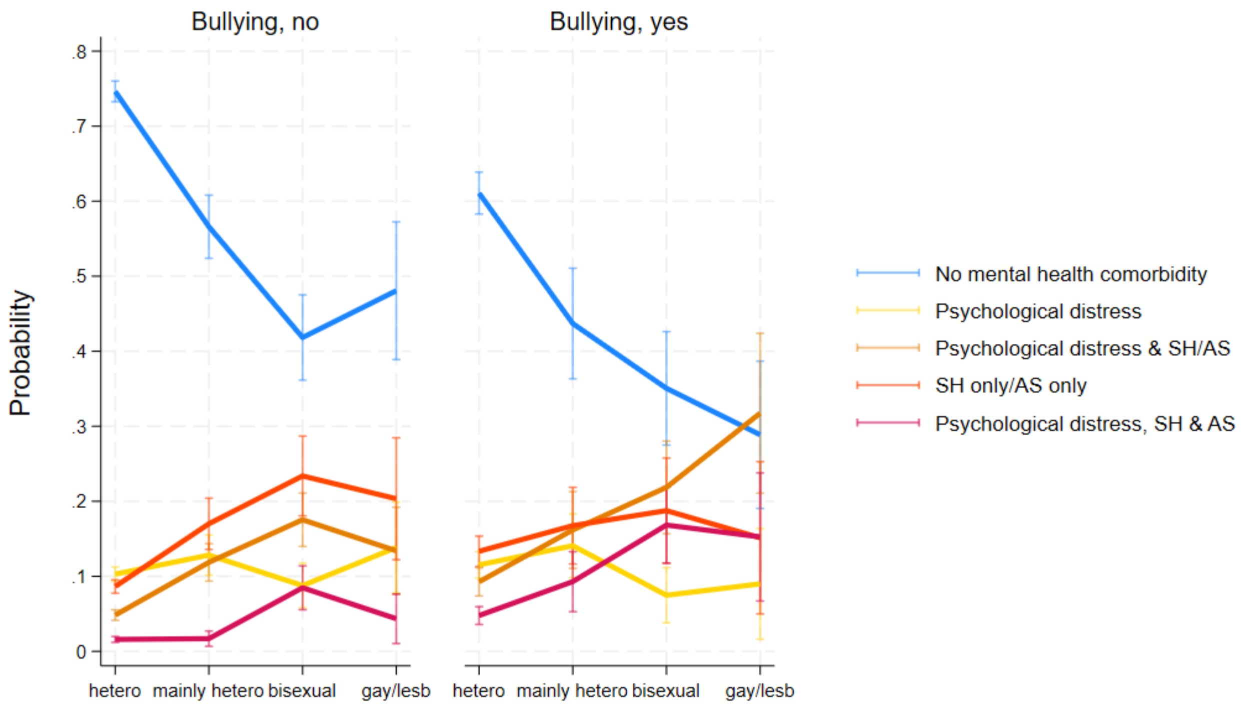

Footnote: Hetero: heterosexual, mainly hetero: mainly heterosexual, SH: Self-harm, AS: Attempted suicide

Probabilities are based on estimates from multinomial logistic regression models including interaction terms between sexual identity and bullying.

Supplemental Table 6. Comparison of key variables between individuals having complete data with the imputed sample

Variable		Complete cases N=8,136 N (%)	Imputed Data N=9,767 (%)
**Bullying**	No	6,552 (78.3)	78
	Yes	1,816 (21.7)	22
**Psychological distress**	No	7,472 (78.3)	78
	Yes	2,066 (21.7)	22
**Self-harm**	No	7,316 (77.5)	77
	Yes	2,122 (22.5)	23
**Attempted suicide**	No	8,830 (92.7)	93
	Yes	695 (7.3)	7

Note: Sex, ethnicity, parental income and sexual identity was available for all participants in the eligible sample
